# Targeted lateral positioning decreases lung collapse and overdistension in COVID-19-associated ARDS

**DOI:** 10.1186/s12890-021-01501-x

**Published:** 2021-04-24

**Authors:** Mikuláš Mlček, Michal Otáhal, João Batista Borges, Glasiele Cristina Alcala, Dominik Hladík, Eduard Kuriščák, Leoš Tejkl, Marcelo Amato, Otomar Kittnar

**Affiliations:** 1 Institute of Physiology, First Faculty of Medicine, Charles University, Prague, Czech Republic; 2 Department of Anaesthesiology, Resuscitation and Intensive Medicine, First Faculty of Medicine, Charles University and General University Hospital in Prague, Prague, Czech Republic; 3 Pulmonology Division, Cardiopulmonary Department, Heart Institute, University of São Paulo, São Paulo, Brazil

**Keywords:** Acute respiratory distress syndrome, Coronavirus disease, Mechanical ventilation, Ventilator-induced lung injury, Positive end-expiratory pressure, Body position

## Abstract

**Background:**

Among the challenges for personalizing the management of mechanically ventilated patients with coronavirus disease (COVID-19)-associated acute respiratory distress syndrome (ARDS) are the effects of different positive end-expiratory pressure (PEEP) levels and body positions in regional lung mechanics. Right-left lung aeration asymmetry and poorly recruitable lungs with increased recruitability with alternating body position between supine and prone have been reported. However, real-time effects of changing body position and PEEP on regional overdistension and collapse, in individual patients, remain largely unknown and not timely monitored. The aim of this study was to individualize PEEP and body positioning in order to reduce the mechanisms of ventilator-induced lung injury: collapse and overdistension.

**Methods:**

We here report a series of five consecutive mechanically ventilated patients with COVID-19-associated ARDS in which sixteen decremental PEEP titrations were performed in the first days of mechanical ventilation (8 titration pairs: supine position immediately followed by 30° *targeted lateral* position). The choice of lateral tilt was based on X-Ray. This *targeted lateral* position strategy was defined by selecting the less aerated lung to be positioned up and the more aerated lung to be positioned down. For each PEEP level, global and regional collapse and overdistension maps and percentages were measured by electrical impedance tomography. Additionally, we present the incidence of lateral asymmetry in a cohort of forty-four patients.

**Results:**

The *targeted lateral* position strategy resulted in significantly smaller amounts of overdistension and collapse when compared with the supine one: less collapse along the PEEP titration was found within the left lung in targeted lateral (*P* = 0.014); and less overdistension along the PEEP titration was found within the right lung in targeted lateral (*P* = 0.005). Regarding collapse within the right lung and overdistension within the left lung***: ***no differences were found for position. In the cohort of forty-four patients, ventilation inequality of > 65/35% was observed in 15% of cases.

**Conclusions:**

Targeted lateral positioning with bedside personalized PEEP provided a selective attenuation of overdistension and collapse in mechanically ventilated patients with COVID-19-associated ARDS and right-left lung aeration/ventilation asymmetry.

**Trial registration:**

Trial registration number: NCT04460859

## Background

Most critically ill patients with coronavirus disease (COVID-19) [1] develops acute respiratory distress syndrome (ARDS), needs mechanical ventilation for prolonged time, and exhibits high mortality [2]. In a large cohort study with critically ill patients with COVID-19 referred for intensive care unit (ICU) admission [2], positive end-expiratory pressure (PEEP) levels were higher than those reported for the management of moderate-to-severe ARDS in the pre–COVID-19 era; and, along with high fraction of inspired oxygen (F_I_O_2_) and low partial pressure of arterial oxygen ratio (PaO_2_/F_I_O_2_) at ICU admission, were an independent factor associated with high mortality. Lung heterogeneity, hypoxemia disproportional to mechanics, right-left lung aeration/ventilation asymmetry [3], and poorly recruitable lungs with increased recruitability with alternating body position between supine and prone [4] have been reported. However, real-time effects of changing body position and PEEP on regional overdistension and collapse, in individual patients, remain largely unknown and not timely monitored.

Lung collapse usually predominates within the most dependent units where the transpulmonary pressure (*P*_L_ = airways pressure − pleural pressure) is the lowest, while lung overdistension predominates within the most nondependent ones where the *P*_L_ is the highest. When there is right-left lung heterogeneity of collapse and overdistension, as in many patients with COVID-19-associated ARDS, a targeted lateral positioning strategy is conceivable: by one-sided lateral position, the lung with more collapsed units in supine position can be positioned gravity-nondependent (mostly) and, conversely, the lung with more overdistended units in supine position can be positioned gravity-dependent. Such targeted lateral position, by which *P*_L_ becomes larger in the nondependent units and smaller in the dependent ones, may afford simultaneous regional/selective recruitment and relief of overdistension effects.

The aim of this study was to individualize PEEP and body positioning in order to reduce lung collapse and overdistension. We hypothesized that targeted lateral positioning with bedside personalized PEEP would provide a selective attenuation of overdistension and collapse in mechanically ventilated patients with COVID-19-associated ARDS and right-left lung aeration/ventilation asymmetry.

## Methods

Patients with COVID-19-associated ARDS in the first days of mechanical ventilation were included. ARDS was defined according to the Berlin definition [5]. COVID-19 was confirmed by positive nasopharyngeal polymerase chain reaction for SARS-CoV-2. Patients were excluded in case of a contraindication to electrical impedance tomography (EIT): pacemaker, implantable defibrillator, skin lesion.

The design was a prospective observational study. The settings were the ICU of the Department of Anaesthesiology, Resuscitation and Intensive Medicine, First Faculty of Medicine, Charles University, General University Hospital in Prague, Czech Republic; and the ICU of the Pulmonology Division, Cardiopulmonary Department, Heart Institute, University of São Paulo, São Paulo, Brazil. All the experiment protocol for involving humans was in accordance to guidelines of national/international/institutional or Declaration of Helsinki. The study was approved by the Ethics Committee of the General University Hospital, Prague, and by the Ethics Committee of the Heart Institute, University of São Paulo, São Paulo.

We used EIT [6] with decremental PEEP titration algorithm (PEEP_EIT-titration_), which provides information on regional overdistension and collapse [7, 8], to individualize PEEP and body position aiming to minimize ventilator-induced lung injury (VILI) mechanisms, namely collapse and overdistension. Electrical impedance tomography (Enlight 1800; Timpel SA, Brazil) is a noninvasive, radiation-free, real-time imaging method that measures global and regional changes in lung volumes [9]. Pulmonary EIT data were recorded at 50 Hz with 32 electrodes equidistantly placed around the circumference of the thorax just below the level of the axilla. Lung collapse and overdistension percentages were determined by comparing each electrical impedance tomography pixel-compliance during PEEP_EIT-titration_ [7, 8]. Each pixel-compliance was determined by dividing tidal impedance change by the variation in pressure during the respiratory cycle. Therefore, overdistension was identified when, for a given pixel, aeration increased and compliance worsened. On the other hand, reversal of collapse was identified if aeration increased and compliance improved.

Sixteen PEEP_EIT-titration_ were performed during the first days of mechanical ventilation in five consecutive patients with COVID-19-associated ARDS in supine immediately followed by *targeted lateral* position (30°). Thus 8 PEEP_EIT-titration_ pairs were obtained. The choice of lateral tilt was based on X-Ray. This *targeted lateral* position strategy was defined by selecting the less aerated lung to be positioned up and the more aerated lung to be positioned down. During all the procedures, the patients were deeply sedated and under muscle paralysis.

Immediately before all PEEP_EIT-titration_, the same lung recruitment maneuver was performed both in supine position and the corresponding targeted lateral position: 2 min of PEEP 24 cmH_2_O and driving pressure of 15 cmH_2_O. The recruitment maneuver allows the measurement of ventilation distribution in all the recruitable pixels. The PEEP_EIT-titration_, which started at a PEEP level of 26 cmH_2_O, were performed with decremental PEEP steps of 2 cmH_2_O until reaching a lower PEEP level set by the clinician.

The EIT data of all PEEP_EIT-titration_ was analyzed in order to quantify the amounts of lung collapse and overdistension at each PEEP step. The lung images were divided in two regions, right and left lung.

It was unclear what percentage of patients with COVID-19-associated ARDS could be potentially suitable for the approach of our study by presenting sufficient right-left lung inequality. To address this issue, we measured the distribution of ventilation to right and left lung from additional 44 patients with COVID-19-associated ARDS. This distribution of ventilation was measured by EIT in supine position. All these patients were ventilated with PEEP set according to the PEEP–F_I_O_2_ table used in the original ARDS Network trial.

### Statistical analysis

The Shapiro–Wilk test was used to test data for normality. The two-way repeated measures ANOVA was used to determine if there was a statistically significant interaction effect between our two within-subjects factors on our continuous dependent variable (i.e., whether a two-way interaction exists). Our continuous dependent variable was % collapse or % overdistension. Our two independent variables were position (supine, lateral) and PEEP [two within-subjects factors]. Simple and main effects were also tested where appropriated. The Bonferroni adjustment for multiple tests was applied for post hoc comparisons. The statistical analyses were conducted with SPSS (version 25; IBM Corp, IBM SPSS Statistics for Windows, Armonk, NY). Individual *P* values to indicate statistical tests’ significance are reported were relevant. Values presented are mean and SEM unless otherwise stated.

## Results

**Fig. 1:**
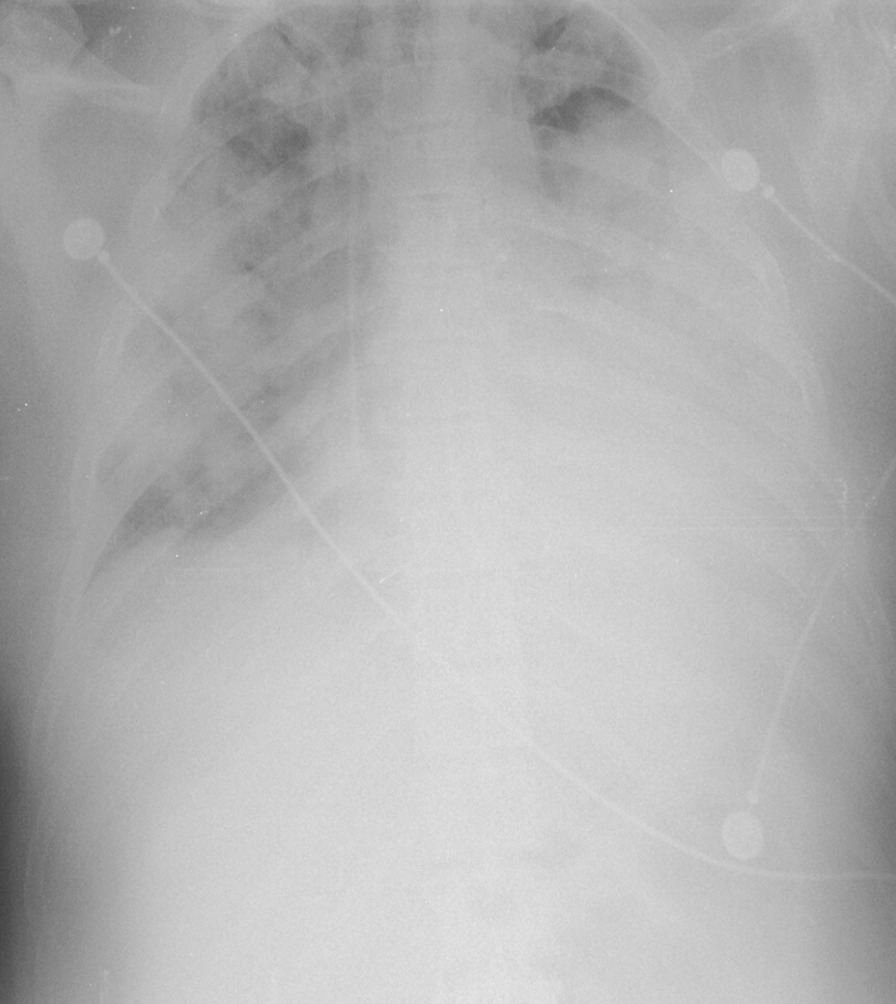
Initial X-Ray in supine body position. The choice of lateral tilt was based on the initial X-Ray that was taken in supine body position. The targeted lateral position strategy was defined by selecting the less aerated lung to be positioned up and the more aerated lung to be positioned down. Please note the left-to-right lung asymmetry present on this initial X-Ray: unequivocally more opacities within the left lung

The five patients (Table 1) that were studied with pairs of PEEP_EIT-titration_ (supine immediately followed by targeted lateral position) exhibited less aeration and ventilation in the left lung (Fig. 1). Thus the right (down) lateral tilt was decided. Below the results of the analyzes of their sixteen PEEP_EIT-titration_ (eight pairs):

**Table 1:** Patients’ characteristics

Sex	Age (yr)	BMI (kg/m^2^)	PaO_2_/F_I_O_2_ ratio (mm Hg)*—supine	PaO_2_/F_I_O_2_ ratio (mm Hg)^†^—lateral	DP (cm HO)^‡^	Respiratory system compliance (ml/cm H_2_O)^§^	Duration of mechanical ventilation at recruitment (days)^¶^
M	44	30,0	396,0	438,0	8,4	35,0	1
F	85	26,8	71,0	N/A	7,2	36,3	2
F	70	24,2	224,0	308,0	6,8	35,0	2
F	82	33,3	521,0	464,0	3,0	60,6	1
M	53	32,1	367,0	441,0	3 7	88,2	9
Mean	66,8	29,3	315,8	412,8	5,8	51,0	3,0
SD	17,9	3,8	172,9	70,8	2,3	23,5	3,4

Definition of abbreviations: BMI = body mass index; PaO_2_/F_I_O_2_ Ratio = ratio of arterial oxygen partial pressure (PaO_2_ in mmHg) to fractional inspired oxygen (F_I_O_2_ expressed as a fraction, not a percentage); DP = driving pressure; N/A = not available

*At the end of the lung recruitment maneuver in supine position

^†^At the end of the lung recruitment maneuver in lateral position

^‡^At PEEP of 12 cm H_2_O

^§^At PEEP of 12 cm H_2_O

^¶^Unavailable because of loss of data

### Collapse-left lung

**Fig. 2:**
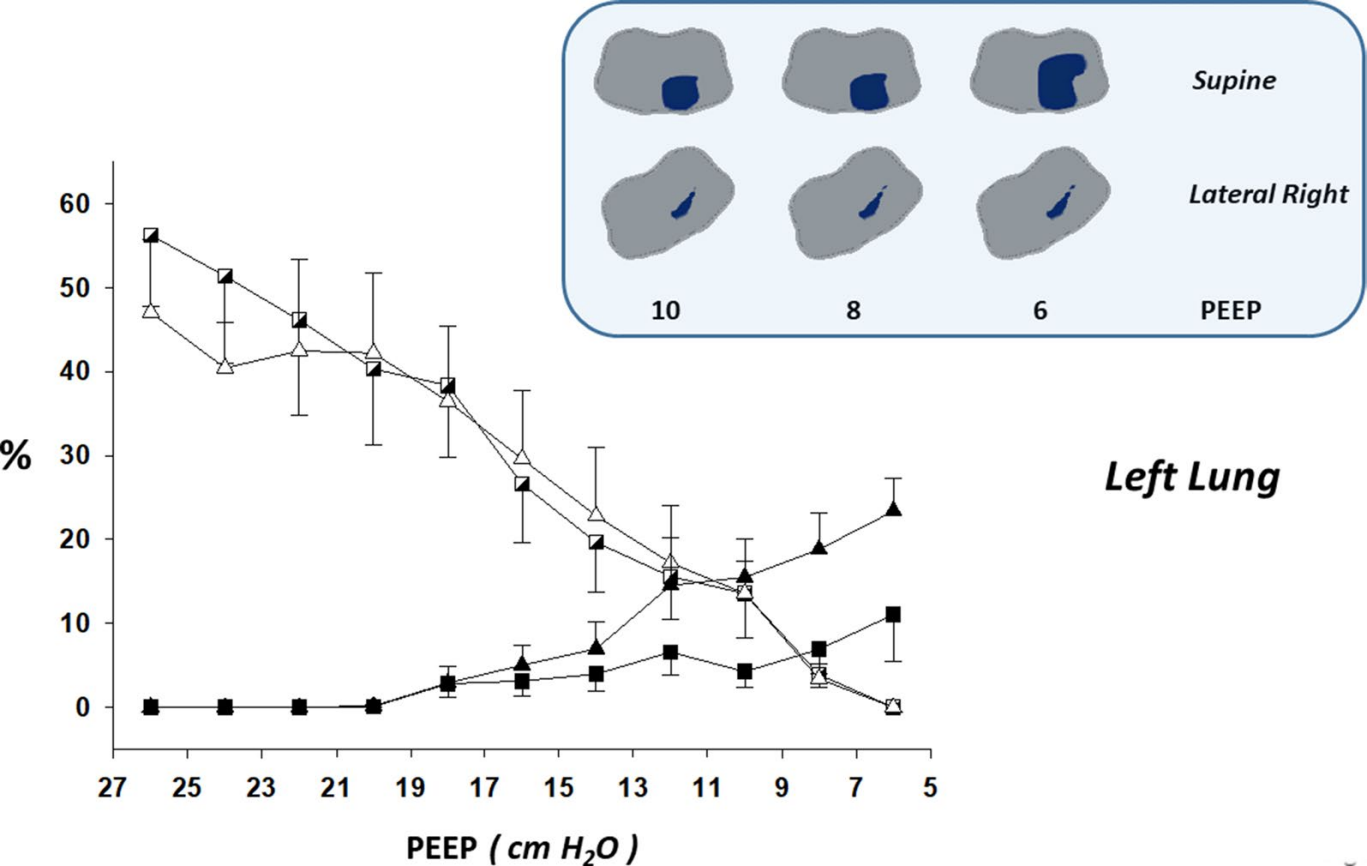
Lung collapse and overdistension by electrical impedance tomography in supine vs. targeted lateral body position within the left lung. Left-to-right lung asymmetry was present on initial X-Ray taken in supine body position: unequivocally more opacities within the left lung. Thus lateral right positioning (30°) was indicated (“targeted”) and performed with the platform-based rotation bed Multicare® (LINET). Line graphs of electrical impedance tomography (EIT)-based estimations of collapse and overdistension during decremental positive end-expiratory pressure (PEEP) titrations (supine vs. targeted lateral body position) are shown (mean ± SEM). Some illustrative and representative EIT images of collapse are also shown: collapsed pixels in purple. Note that the amount of collapsed units within the left lung present in the supine body position was minimized in the lateral right one. X axis: Decremental PEEP levels of the EIT-PEEP titrations. Y axis: Percent of overdistended and collapsed lung units out of the total lung imaged by EIT. Triangle: Supine body position. Square: Targeted lateral body position (lateral right). Black triangle and black square: Percent of collapsed lung units out of the total lung imaged by EIT. White triangle and white semi-filled square: Percent of overdistended lung units out of the total lung imaged by EIT

There was a statistically significant two-way interaction between position (supine vs. targeted lateral) and PEEP (*P* = 0.014; two-way repeated measures ANOVA) in the % of collapse within the left lung: less collapse along the PEEP titration was found within the left lung in targeted lateral (right down) than supine position (Fig. 2). Additionally, when the simple main effects were tested, the following significant differences were found: PEEP 14 (*P* = 0.034), PEEP 10 (*P* = 0.028), PEEP 8 (*P* = 0.019), and PEEP 6 cmH_2_O (*P* = 0.007).

### Overdistension-right lung

**Fig. 3:**
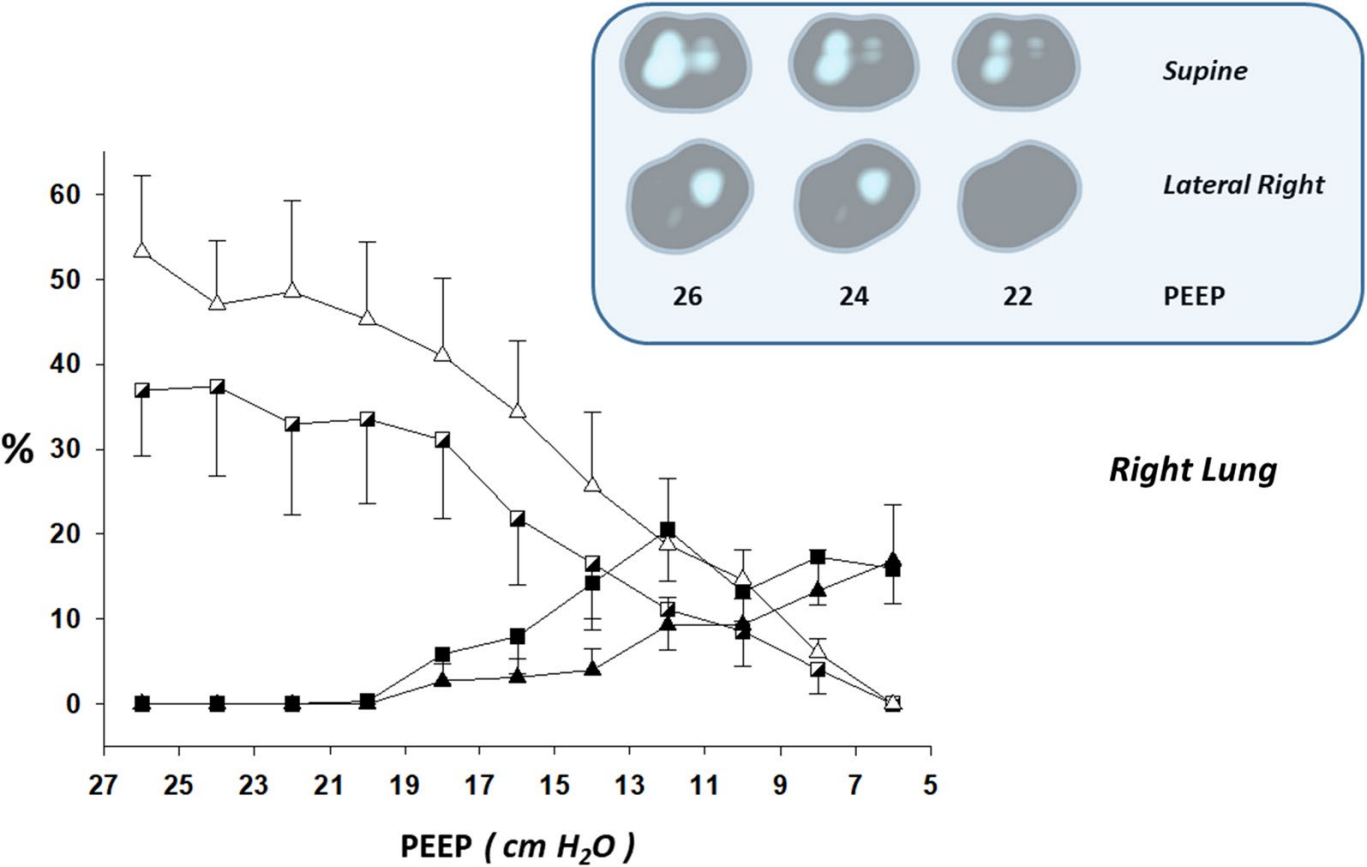
Lung collapse and overdistension by electrical impedance tomography in supine vs. targeted lateral body position within the right lung. Left-to-right lung asymmetry was present on initial X-Ray taken in supine body position: unequivocally more opacities within the left lung. Thus lateral right positioning (30°) was indicated (“targeted”) and performed with the platform-based rotation bed Multicare® (LINET). Line graphs of electrical impedance tomography (EIT)-based estimations of collapse and overdistension during decremental positive end-expiratory pressure (PEEP) titrations (supine vs. targeted lateral body position) are shown (mean ± SEM). Some illustrative and representative EIT images of overdistension are also shown: overdistended pixels in white. Note the asymmetric distribution of overdistension between the right and left lungs (concentration and predominance of overdistension within the right lung); and that the amount of overdistended units within the right lung in the supine body position was minimized in the lateral right one. Also note that the regional distribution of overdistension in the supine body position was much less gravitational-dependent than it is usually present in “typical” acute respiratory distress syndrome. X axis: Decremental PEEP levels of the EIT-PEEP titrations. Y axis: Percent of overdistended and collapsed lung units out of the total lung imaged by EIT. Triangle: Supine body position. Square: Targeted lateral body position (lateral right). Black triangle and black square: Percent of collapsed lung units out of the total lung imaged by EIT. White triangle and white semi-filled square: Percent of overdistended lung units out of the total lung imaged by EIT

There was a marginal two-way interaction between position and PEEP (*P* = 0.073; two-way repeated measures ANOVA). The main effect of position showed a statistically significant difference in the % of overdistension within the right lung: less overdistension along the PEEP titration in targeted lateral (right down) than supine position (*P* = 0.005; Fig. 3). The main effect of PEEP on right lung overdistension showed a statistically significant difference (*P* < 0.0005). Additionally, for many PEEP levels significant *P* values were found in the pairwise comparisons with adjustment for multiple comparisons (Bonferroni).

### Collapse-right lung and overdistension-left lung

No statistically significant differences were found for position.

Regarding the percentage of patients with COVID-19-associated ARDS presenting sufficient right-left lung inequality: the mean of the % of ventilation of the right lung was 54.9 ± 8.2 and the mean of the % of ventilation of the left lung was 45.3 ± 8.1. A right-left lung asymmetrical ventilation of > 65/35% was observed in 15% of these patients. This distribution of ventilation was measured by EIT in supine position and under a mean PEEP level of 11.4 ± 3.1 cmH_2_O (set according to the PEEP–F_I_O_2_ table used in the original ARDS Network trial).

The optimum PEEP of PEEP_EIT-titration_ was defined by the best compromise between pulmonary overdistension and collapse, i.e. the smallest sum of overdistension and collapse. The optimum PEEP was 10.8 ± 5.3 cmH_2_O in supine position and was 11.2 ± 5.9 cmH_2_O in targeted lateral position.

Hemodynamic compromise was not detected for any patients during all procedures of the study.

## Discussion

A major focus of mechanical ventilation for COVID-19 is the avoidance of VILI while facilitating gas exchange via lung-protective ventilation. This is the first description of using EIT with targeted lateral positioning to personalize PEEP in adult patients with COVID-19-associated ARDS. A randomized and controlled trial demonstrated the feasibility and efficacy of a postural recruitment maneuver in children with anesthesia-induced atelectasis [10]. Besides being applied in children with healthy lungs, another difference between the Acosta et al. and our study is the lack of PEEP titrations. Very recently, Zhao et al. reported the use of EIT for individualized ventilation strategy in one patient with COVID-19 [11]. Similarly to the study in children with anesthesia-induced atelectasis [10], a key difference between the Zhao et al. case report and our case series is the lack of a strategy to personalize PEEP during the lateral positioning.

The vertical gradient of *P*_L_, which is mainly due to gravity, changes with body mass and posture [12]. Agostoni and D’Angelo showed that the *P*_L_ gradient increased when body position was changed from supine to lateral position [13]. They demonstrated that lateral position leads to higher *P*_L_ in the most nondependent units and lower *P*_L_ in the most dependent ones. That is mainly because the thoracic right-to-left distance is longer than the anterior–posterior. Thus, generally, lateral positioning increases heterogeneity of P_L_ across the parenchyma, but this depends on PEEP and baseline lung conditions: recent reports in children have shown that an optimized PEEP after lateralization can minimize hyperdistension (maximizing ventilation) in a nondependent, sicker lung, while reasonably keeping functional residual capacity in dependent, healthier lung [14]. Our PEEP_EIT-titration_ seems to be a promising tool to find such personalized PEEP at the bedside.

Another potential beneficial effect of the targeted lateral positioning is an improved ventilation/perfusion matching due to: 1) attenuation of regional overdistension within the more aerated lung and, consequently, less diversion of pulmonary blood flow away from these units; 2) diminution of regional collapse within the less aerated lung. The consequent improvement of oxygenation may be important in these patients to manage their disproportional hypoxemia and “buy time” with minimum additional damage.

Our findings reinforce the importance of timely PEEP titrations [15] tackling the dynamically changing phases of this disease. They suggest the relevance of personalized PEEP adjustments every time body positions are changed. The recommendation of applying nonpersonalized low or high PEEP may lead to insufficient and/or excessive PEEP in terms of protection of VILI [16].

## Conclusions

Targeted lateral positioning with bedside personalized PEEP provided a selective attenuation of overdistension and collapse in mechanically ventilated patients with COVID-19-associated ARDS and right-left lung aeration/ventilation asymmetry.

## Data Availability

The data are with the authors and will be available upon reasonable request. The data is available from the corresponding author: João Batista Borges.

## Abbreviations

COVID-19: Coronavirus disease
ARDS: Acute respiratory distress syndrome
ICU: Intensive care unit
PEEP: Positive end-expiratory pressure
F_I_O_2_: Fraction of inspired oxygen
PaO_2_: Arterial oxygen partial pressure
PaO_2_/F_I_O_2_: Partial pressure of arterial oxygen ratio
*P*_L_: Transpulmonary pressure (*P*_L_ = airways pressure − pleural pressure)
EIT: Electrical impedance tomography
PEEP_EIT-titration_: PEEP titration algorithm using EIT
VILI: Ventilator-induced lung injury

## References

[1] Coronaviridae Study Group of the International Committee on Taxonomy of V: The species Severe acute respiratory syndrome-related coronavirus: classifying 2019-nCoV and naming it SARS-CoV-2. Nature Microbiol 2020, **5**(4):536–544. 10.1038/s41564-020-0695-z PMC7095448 32123347

[2] Grasselli G, Greco M, Zanella A, Albano G, Antonelli M, Bellani G, Bonanomi E, Cabrini L, Carlesso E, Castelli G (2020). Risk factors associated with mortality among patients with COVID-19 in intensive care units in lombardy Italy. JAMA Internal Med.

[3] Bhatraju PK, Ghassemieh BJ, Nichols M, Kim R, Jerome KR, Nalla AK, Greninger AL, Pipavath S, Wurfel MM, Evans L (2020). Covid-19 in critically Ill patients in the seattle region - case series. N Engl J Med.

[4] Pan C, Chen L, Lu C, Zhang W, Xia JA, Sklar MC, Du B, Brochard L, Qiu H (2020). Lung recruitability in COVID-19-associated acute respiratory distress syndrome: a single-center observational study. Am J Respir Crit Care Med.

[5] Force ADT, Ranieri VM, Rubenfeld GD, Thompson BT, Ferguson ND, Caldwell E, Fan E, Camporota L, Slutsky AS (2012). Acute respiratory distress syndrome: the Berlin definition. JAMA.

[6] Borges JB, Cronin JN, Crockett DC, Hedenstierna G, Larsson A, Formenti F (2020). Real-time effects of PEEP and tidal volume on regional ventilation and perfusion in experimental lung injury. Intensive Care Med Exp.

[7] Costa EL, Borges JB, Melo A, Suarez-Sipmann F, Toufen C, Bohm SH, Amato MB (2009). Bedside estimation of recruitable alveolar collapse and hyperdistension by electrical impedance tomography. Intensive Care Med.

[8] Fumagalli J, Santiago RRS, Teggia Droghi M, Zhang C, Fintelmann FJ, Troschel FM, Morais CCA, Amato MBP, Kacmarek RM, Berra L (2019). Lung recruitment in obese patients with acute respiratory distress syndrome. Anesthesiology.

[9] Victorino JA, Borges JB, Okamoto VN, Matos GF, Tucci MR, Caramez MP, Tanaka H, Sipmann FS, Santos DC, Barbas CS (2004). Imbalances in regional lung ventilation: a validation study on electrical impedance tomography. Am J Respir Crit Care Med.

[10] Acosta CM, Volpicelli G, Rudzik N, Venturin N, Gerez S, Ricci L, Natal M, Tusman G (2020). Feasibility of postural lung recruitment maneuver in children: a randomized, controlled study. Ultrasound J.

[11] Zhao Z, Zhang JS, Chen YT, Chang HT, Hsu YL, Frerichs I, Adler A (2021). The use of electrical impedance tomography for individualized ventilation strategy in COVID-19: a case report. BMC Pulm Med.

[12] D'Angelo E, Bonanni MV, Michelini S, Agostoni E (1970). Topography of the pleural pressure in rabbits and dogs. Respir Physiol.

[13] Agostoni E, D'Angelo E, Bonanni MV (1970). The effect of the abdomen on the vertical gradient of pleural surface pressure. Respir Physiol.

[14] Schibler A, Henning R (2002). Positive end-expiratory pressure and ventilation inhomogeneity in mechanically ventilated children. Pediat Crit Care Med.

[15] van der Zee P, Somhorst P, Endeman H, Gommers D (2020). Electrical impedance tomography for positive end-expiratory pressure titration in COVID-19-related acute respiratory distress syndrome. Am J Respir Crit Care Med.

[16] Fan E, Beitler JR, Brochard L, Calfee CS, Ferguson ND, Slutsky AS, Brodie D (2020). COVID-19-associated acute respiratory distress syndrome: is a different approach to management warranted?. Lancet Respir Med.

